# Commencement Exercises of the Medical Department of the University of Buffalo

**Published:** 1863-03

**Authors:** 


### EDITORIAL DEPARTMENT.

### COMMENCEMENT EXERCISES OF THE MEDICAL DEPARTMENT OF THE UNIVERSITY OF BUFFALO.

The Annual Commencement exercises of the Medical Department of the Univerity of Buffalo were held at the Opera House, and were attended by a large number of citizens.

The following gentlemen received the degree of *Doctor in Medicine:*

Heman Potter Babcock, Buffalo, Erie County, N. Y.
Gilbert Birdsall, Butternuts, Otsego County, N. Y.
Andrew Thomas Dunn, Brockville, Leeds County, C. W.
James Usher Babcock, Elmira, Chemung County, N. Y.
Howard Wellington Vickery, Darien, Genesee County, N. Y.
George Dean, Sullivan, Chemung County, N. Y.
Edwin Booth, Jelloway, Knox County, Ohio.
Benjamin Booth Ross, Belleville, Hastings County, C. W.
Nehemiah Osborn, Belleville, Hastings County, C. W.
Robert Wickliffe Gifford, Ashtabula, Ashtabula County, Ohio.
John Henry Tanner, Hartford, Cortland County, N. Y.
James Dormid, De Ruyter, Madison County, N. Y.
Asa John White, Mecklenberg, Schuyler County, N. Y.
Dascomb Allen Farrington, East Aurora, Erie County, N. Y.
Andrew Jackson Scott, Loudonville, Ashland County, Ohio.
Orlando L. Abbey, Union Mills, Erie County, Pa.
John Coventry, Clarkville, Kent County, C. W.
Earl Byron Lonusbury, Bethany Mills, Genesee County, N. Y.
Fletcher Miller Follett, Machias, Cattaraugus County, N. Y.
Daniel Winter, Pekin, Niagara County, N. Y.
David Hershey, Fort Erie, C. W.
Edwin Parker Moore, Jamestown, Chautauqua County, N. Y.
Jeremiah Andrews, Sugar Grove, Warren County, Pa.
Edward H. Beaman, Ridgeway, C. W.

The following Theses were deemed by the Faculty as worthy of honorable mention, viz:

A Thesis on the "Uterus and its Appendages," by A. J. Scott, of Loudonvile, Ashland County, Ohio, and a Thesis on "Arsenic," by A. T. Dunn, of Brockville, Leeds County, C. W.

The Valedictory Address was delivered by Mr. Benjamin B. Ross, and was very appropriate for the occasion. After referring to the sundering of old connections and the sadness which was felt on separating from college associates, and also the toils and anxieties of student life, the brighter vision of the future, was portrayed, only claiming for himself and his class, that they had passed the vestibule of the temple of professional knowledge, the temple itself as yet untraversed.

The sincere and heartfelt thanks of the class were extended to the Professors of the College, and the assurance given, that the pathway of life could have no pleasanter recollections, than wonld be furnished by their connections with the institution from which they graduated.

The Address was closed by very hearty good wishes for all, and a parting farewell.

Professor Rochester delivered the charge to the graduates, which we take pleasure in publishing.

After the public exercises, the Faculty invited the council, curators and graduating class to a supper, which was served at the American Hotel. When the inner man had been satisfied, Prof. Eastman made remarks upon the pride of the Faculty in the yearly fruit of the College, the pleasant associations of the past winter, and the hearty wishes of the Faculty for the success and prosperity of each member of the graduating class.—Excused himself from making a speech, and proposed the Orator of the evening.

Prof. Rochester made brief remarks in response, and called upon Prof. White for a speech.

Prof. White spoke of the necessity of harmony in the Faculty of the College, and said he had noticed that day something which looked like a conspiracy—that he had seen some of the Faculty with a paper they were concealing from the Dean. He then read the following note:

BUFFALO, February 24, 1863.

Prof. Sandford Eastman, M. D., Dean of the Faculty of the Medical Department of the University of Buffalo:

Dear Sir:—The undersigned, fellow members of the Faculty of the Medical Department of the University of Buffalo beg to assure you of their high estimate of your efficient and valuable services, in behalf of the interests of the College, during the session now drawing to a close.

As a slight testimonial of our appreciation of your unwearied and self-sacrificing labors, in advancing the welfare of the School in which we are mutually interested, we request your acceptance of the accompanying Case of Instruments, sincerely hoping that you may find them useful in a department of Surgical practice in which you have recently achieved a signal success.

With the best wishes for your future welfare,

We remain very truly,
Your friends and colleagues,
JAMES P. WHITE, GEORGE HADLEY,
THOMAS F. ROCHESTER, WILLIAM H. MASON,
J. R. LOTHROP.

Prof. Eastman said, "there are times when silence speaks louder than words. Was never more surprised in my life, and have no language to express my emotions. In the embarrassments of such a surprise can only express my thanks to my colleagues for this very unexpected expression of approval."

Prof. White remarked upon the existence and merits of the *Buffalo Medical and Surgical Journal*, and after recommending it to the graduating class, called upon the Editor for a speech.

Dr. Miner said that he always held himself ready to speak for the Buffalo Medical and Surgical Jurnal upon all appropriate occasions, being well acquainted with its history and the forces by which it had been instituted and maintained. After saying, that he hoped the graduating class would be able to continue their acquaintance with the Professors of the University of Buffalo, through the columns of the Journal, he had little else which he desired to say of the Buffalo Journal in particular, but by a little extension of privilege would speak upon the power and influence of the Periodical Medical Press in general. He desired to do this for the benefit of the young men who are just entering the field of medical knowledge, that they may not neglect the longest lever of professional power, by which alone they may move the medical world. Medical colleges and their professors are a great power, and wield a wide influence, especially over medical students, and thus also over medical men. We are taught by them however in our pupilage the established and universally acknowledged principles of medicine, surgery, and the collateral sciences, while in actual practice we consult our written guides; then it is, that those only who write, are those who teach. Standard medical books are also a great power in the profession, but they are also dependent in great degree upon the Periodical Medical Press for both the material of which they are composed, and their adoption by the profession. Again, many physicians do not carefully read the voluminous works in medicine, while it is believed that all are more or less acquainted with periodical medical literature.— These suggestions are made that this graduating class may not be unmindful of the importance to them of medical journals. You may be of value to the journals, but the journals are of vastly greater value to you. They are practically the only available medium of communication with the profession; through them you will learn much of what you will know, and whatever of any professional importance is ever known of you, will be through such medium. Your attention is therefore called to the periodical medical press, as the all-powerful influence in the profession.

Mr. Shepherd, member of the Council, made brief remarks upon the origin, growth, present condition and future prospects of the University. Expected that the University of Buffalo would yet become worthy the name. Spoke of the necessity of time in the growth of such institutions, and the endowment of professorships necessary to the full realization of the expectations of its friends, which comprise especially the older and more influential citizens of Buffalo. Had been a member of the Council from the first, and had watched the progress of the Medical Department with deep interest.

Prof. Lothrop was called upon, and spoke upon the character of the Chair assigned to him, the matter of fact, statistical, and dry subjects he had to deal with. Hoped the young men would remember that stimulants at length became sedatives, and that cathartics were often necessary or useful, after over doses. *Purgation* was not so agreeable as stimulation, but often much more beneficial. Remarked also upon the great amount of teaching they had received without as yet any opportunity for practical application, and regarded the bed-side as, after all, the only true place to learn the value of medicines. Though not himself over credulous as to the value or beneficial influence of medicine over disease, still we could not disregard the accumulated experience of the profession; hoped it would be remembered however that medicines, as the instruments with which we treat disease, are double-edged, and cut both ways.

Sentiments and responses were still sufficiently numerous and varied; many very pleasant and smart speeches were made, but at length the intellectual supply somewhat degenerated, though the exercises for the most part were exceedingly pleasant and interesting.

We have had handed us a copy of the resolutions presented to the Professors of the University, by the graduating class, complimentary of the School, and expressive of thanks for the ability and efficiency with which the duties of each professorship had been discharged. Since these resolutions have been handed the daily press for publication, and since we regard the College and Professors as above requiring any especial recommendation or endorsement; also considering the space already devoted to a report of College commencement, we hope we may be excused for the omission of these resolutions from our pages, not, however, without saying, that these tokens of appreciation, on the part of the Class, when made to their teachers privately, may be highly appropriate and becoming.

